# Improving Family, Carer and Community Professional Involvement in Patient Care for Individuals Admitted to a Psychiatric Intensive Care Unit

**DOI:** 10.1192/bjo.2025.10459

**Published:** 2025-06-20

**Authors:** Kirsty Ward, Natasha Treagust, Suveera Prasad

**Affiliations:** Rotherham, Doncaster and South Humber NHS Foundation Trust, Doncaster, United Kingdom

## Abstract

**Aims:** To assess and improve the consistency of family, carer and community team involvement in our MDT (multidisciplinary team) ward rounds on a PICU (Psychiatric Intensive Care Unit).

**Methods:** Baseline date involved retrospective analysis of EPR (electronic patient records) reviewing the documentation of 27 admissions over a six-month period (February to August 2024). We recorded details of demographics, diagnosis and length of stay. We reviewed MDT documentation for a maximum of eight weeks to ascertain invites, attendees and whether family/carers had been contacted within three days of their first MDT as this was an agreed expectation. A total of 101 MDTs were reviewed.

Following baseline data collection we made the following changes:

Circulation of expected standards regarding family involvement.

Change in format of our daily handover document to include details of important family/carer contacts and most recent recorded contact.

Use of a standardised MDT documentation proforma to improve accuracy of written records.

**Results:** Baseline data: The majority of our patients were male (74.1%), White British (74.1%), English speakers (88.9%), from the local area (81.5%) and had a diagnosis of a schizophrenic, schizotypal or delusional disorder (51.9%). The average age was 35 years and length of admission 35.5 days.

59.3% of admissions had documented contact with relevant family/carer within three days of their first MDT review.

On average relevant family/carers/professionals were invited to 61.4% of MDTs and attended 45.3% of the time.

On average MDT attendance reduced over time from 48.1% in week one to 33.3% in week eight.

Re-audit data: We are in the process of prospective re-audit between the period of January to March 2025. Thus far we have reviewed the records for 9 patients and analysed data entry of 18 individual MDTs.

100% of admissions had documented contact with relevant family/carer within three days of their first MDT review.

On average relevant family/carers/professionals were invited to 81.5% of MDTs and attended 59.3% of the time.

The MDT documentation proforma use was 61.5%.

We expect to have a larger data set as collection is ongoing.

**Conclusion:** Quality and accuracy of documentation is likely to have underestimated the frequency of contact with carers, particularly in the baseline data.

Timely, regular and meaningful liaison with family, carers and community professionals improves outcomes and quality of care for PICU patients. Simple interventions and reinforcement of expected practice can improve frequency and consistency of patient and carer involvement.

